# Total Knee Arthroplasty in Patients with Prior Femoral and Tibial Fractures: Outcomes and Risk Factors for Surgical Site Complications and Reoperations

**DOI:** 10.1111/os.12610

**Published:** 2020-01-20

**Authors:** Xing‐shan Wang, Yi‐xin Zhou, Hong‐yi Shao, De‐jin Yang, Yong Huang, Fang‐fang Duan

**Affiliations:** ^1^ Department of Orthopaedics Beijing Jishuitan Hospital, Fourth Clinical College of Peking University Beijing China; ^2^ Clinical Epidemiology Research Center Beijing Jishuitan Hospital, Fourth Clinical College of Peking University Beijing China

**Keywords:** Complications, Osteoarthritis, Patella baja, Post‐traumatic, Total knee arthroplasty

## Abstract

**Objective:**

To investigate the outcomes of total knee arthroplasty (TKA) in patients with a prior femoral or tibial fracture, and identify the risk factors for surgical site complications and reoperations.

**Methods:**

Seventy‐one TKAs performed in 71 patients with a prior tibial or femoral fracture between January 2005 and December 2016 were reviewed retrospectively. Forty males (40 knees) and 31 females (31 knees) were included. The mean age at the time of TKA was 59.2 (range, 29–83) years. Outcomes were assessed using the Knee Society score before surgery and at the final follow‐up visit. The patients' satisfaction rates were evaluated. Complications and reoperations were recorded by clinical and radiographic assessment. Logistic regression analysis was used to identify the risk factors for surgical site complications and reoperations.

**Results:**

The median follow‐up period was 4.7 (range, 3.2–7.1) years. The median knee range of motion increased from 90° preoperatively to 110° at the latest follow‐up. The Knee Society knee score and function score improved from 35 (30, 40) and 40 (30, 50) to 90 (82, 93) and 90 (65, 100), respectively. The degree of overall satisfaction after TKA surgery was very satisfied in 41 patients, satisfied in 20 patients, neutral in four patients, dissatisfied in four patients, and very dissatisfied in two patients. The overall satisfaction (very satisfied and satisfied) rate was 85.9% (61 knees). Twelve knees (16.9%) had 19 surgical site complications. Six knees (8.3%) underwent reoperations, including one revision due to periprosthetic joint infection, one debridement and implant retention for superficial infection, two debridements for delayed wound healing, one open reduction and internal fixation for supracondylar fracture, and one re‐fixation and bone grafting for hardware failure after a combined femoral shaft osteotomy and TKA. Preoperative patella baja was diagnosed in 12 knees, and was identified as a risk factor for surgical site complications and reoperations.

**Conclusions:**

TKA for post‐fracture osteoarthritis significantly relieved pain and improved function, but the incidence of surgical site complications and reoperations was high. Preoperative patella baja was a risk factor for surgical site complications and reoperations.

## Introduction

Post‐traumatic osteoarthritis (PTOA) of the knee is defined as the development of arthritis following an acute traumatic episode commonly associated with significant ligamentous injury or intra/extra‐articular fracture^1^. PTOA represents 9.8% of the overall prevalence of symptomatic knee osteoarthritis, costing an estimated $40 bn in direct and indirect costs^2^. Femoral and tibial fractures represent some of the major causes of PTOA of the knee^3^. PTOA may be caused by either intra‐articular fractures, which result in direct ligament and osteochondral injury, and cause joint instability and incongruity, or secondary to malunion of extra‐articular fractures around the knee, which alters weight‐bearing axis of the lower limb, thereby increases the joint stress and accelerates the joint degeneration. Patients sustaining distal femur or proximal tibia fractures are around twice as likely to require total knee arthroplasty (TKA) than patients with soft‐tissue injuries^4^, ^5^.

Due to previous surgery, retained hardware, bone defects, and the extra‐articular angular deformity created by a fracture, TKA for PTOA is technically demanding even for experienced surgeons. Patients with PTOA are also susceptible to higher rates of complications, including aseptic mechanical failure, periprosthetic joint infection, wound healing problems, and higher rates of reoperation compared with TKA performed for atraumatic osteoarthritis^6^, ^7^. Bala *et al*.^6^ evaluated the impact of PTOA *versus* primary osteoarthritis on postoperative outcomes after TKA in a large database of Medicare patients, and found PTOA patients had higher incidence of periprosthetic infection (OR 1.72, *P* < 0.001), cellulitis or seroma (OR 1.19, *P* < 0.001), knee wound complications (OR 1.80, *P* < 0.001), TKA revision (OR 1.23, *P* = 0.01), and arthrotomy/incision and drainage (OR 1.55, *P* < 0.001).

Previous literature has found several risk factors of unsatisfactory outcomes after TKA for PTOA. Shearer *et al*.^8^ noted that the location of post‐traumatic deformity and compromise of the soft‐tissue envelope influence the pain and functional outcomes of TKA for PTOA. Isolated articular deformities have the largest improvement in pain and function while patients with combined tibial and femoral deformities as well as patients with soft‐tissue compromise experienced poor outcomes. Ge *et al*.^9^ reported that patients with previous site‐specific fractures suffered higher surgical site complications (22% *vs* 4.4%) and 90‐day readmissions (14.8% *vs* 2.2%) after conversion TKA than patients with previous soft‐tissue knee trauma. El‐Galaly *et al*.^10^ found an increased risk of early and medium‐term revision of TKAs due to previous fractures in the distal femur and/or proximal tibia. To the best of our knowledge, there is a scarcity of studies pertaining to the risk factors for surgical site complications and reoperations after TKA in patients with PTOA secondary to prior femoral and tibial fractures.

The purpose of the present study was to: (i) evaluate the clinical outcomes after TKA following femoral and tibial fractures; (ii) investigate the incidence of surgical site complications and reoperations; and (iii) identify the risk factors for surgical site complications and reoperations after TKA in patients with PTOA secondary to prior femoral and tibial fractures.

## Methods

### Inclusion and Exclusion Criteria

Patients were selected using the following inclusion criteria: (i) the primary diagnoses of the patients were post‐traumatic osteoarthritis secondary to tibial or femoral fracture; (ii) patients were treated by TKA; (iii) patients had intact clinical and radiographic data; (iv) patients had a minimum 2‐year follow‐up period. The exclusion criteria included: (i) patients had a history of rheumatic diseases; (ii) patients lacked complete medical and radiographic records; or (iii) patients had a less‐than‐2‐year follow‐up period.

### Study Design

This retrospective study was approved by our institutional review board. Patients who underwent TKA for PTOA in our institution between January 2005 and December 2016 were reviewed. Informed consent was provided by all participants.

### Patient Characteristics

A total of 85 consecutive patients (86 knees) were initially identified. After applying the inclusion criteria, 71 cases (71 knees) were eligible for our study. There were 40 males (40 knees) and 31 females (31 knees). The mean body mass index (BMI) was 26.4 (range, 20.5–35.1). The mean age at the time of TKA was 59.2 (range, 29–83) years (Table 1).

**Table 1:** Demographic and Clinical Characteristics of the Patients

Variable	Value
Gender, n	
Female	40
Male	31
Age (years), mean ± SD	59.2 ± 10.7
BMI*, mean ± SD	26.5 ± 3.3
Preoperative ROM(°), median (IQR^#^)	90.0 (65.0, 100.0)
Operation time(minutes), median (IQR^#^)	110.0 (80.0, 140.0)
Follow‐up period (years), median(IQR^#^)	4.7 (3.2, 7.1)
Site of prior fracture, n	
Femur	32
Tibia	35
Combined	4
Location of prior fracture, n	
Metaphysis	19
Diaphysis	33
Intra‐articular	19
Degree of coronal deformity, n	
≤10°	36
10°–20°	24
≥20°	11
Patella baja, n	
No	59
Yes	12
Hardware retained, n	
No	53
Yes	18
Surgical history, n	
No	45
Yes	26

Note: *, body mass index. ^#^, interquartile range.

### Location of Prior Fracture

Of the 71 fractures, there were 32 femoral fractures, 35 tibial fractures, and four combined femoral and tibial fractures. The location of prior fracture was the metaphysis in 19 fractures, diaphysis in 33 fractures, and there was intra‐articular involvement in 19 fractures.

### Initial Treatment of Prior Fracture

Twenty‐three knees had undergone open reduction and internal fixation for prior fractures, 18 of which had retained hardware prior to the TKA. Two knees had undergone debridement and cast immobilization for open fractures, and 46 knees had received nonoperative treatment.

### Coronal Deformities

The coronal deformities of the lower limb were measured on full‐length, weight‐bearing radiographs by measuring the hip‐knee‐ankle alignment (HKA) angle, and then were classified as: type A,≤10° of varus/valgus deformity; type B, 11 to 20° of varus/valgus deformity; type C, >20° of varus/valgus deformity. The varus/valgus deformity was type A in 36 knees, type B in 24 knees, and type C in 19 knees.

### Patella Height

The Blackburne–Peel ratio^11^ was applied to identify patella baja. Patella baja was diagnosed if the ratio was less than 0.54, and was identified in 12 knees (Table 1).

### Surgical Technique

All TKAs were cemented and performed by senior surgeons experienced in primary and revision TKA surgery. A general anesthetic or a spinal anesthetic was chosen depending upon the patient's ASA grade and medical history. Femoral and sciatic nerve blocks were used additionally for postoperative pain relief. Then the patients were placed on supine position.

A medial parapatellar approach was used in 64 knees, while a lateral approach was used in seven knees due to a previous surgical incision.

A measured resection technique was utilized for all TKA procedures. The aim was to obtain neutral mechanical alignment and balanced flexion/extension gaps. Navigation‐assisted TKA was performed in 10 knees for partially retained hardware, extra‐articular angular deformity, or canal sclerosis, which prevented the use of an intramedullary guide on the femoral side. Fifty‐nine posterior‐stabilized (PS) prostheses, six cruciate‐retaining (CR) prostheses, five condylar‐constrained knee (CCK) prostheses, and one rotating hinge prosthesis were used. Long‐stemmed tibial components were used to ensure the implant fixation in eight cases due to severe bone defect secondary to tibial plateau fracture.

### Clinical and Radiographic Assessment

Clinical and radiographic assessment was performed before TKA and at each follow‐up postoperatively. Radiological evaluation was performed using standard standing anterior–posterior and lateral views, and full length radiograph of the lower extremity. The following indicators were retrospectively recorded.

### Knee Society Score (KSS)

Knee function was evaluated before surgery and at each follow‐up using the Knee Society score, which consists of knee score and function score, each with a maximum of 100 points. The knee score rates the knee joint based on pain, stability and range of motion, with deduction for flexion contracture, extension lag and malalignment. The function score rates the patient's ability to walk and climb stairs, with deduction for aids.

### Overall Satisfaction

Patients were asked to grade their degree of overall satisfaction with the results of TKA at their last follow‐up visit. The main question was how satisfied (very satisfied, satisfied, neutral, dissatisfied, or very dissatisfied) patients were with the results of their TKA^12^. Patients who were satisfied or very satisfied were classified as “overall satisfied”, and patients who were neutral, dissatisfied, or very dissatisfied were classified as “overall dissatisfied”.

### Surgical Site Complications and Reoperations

Surgical site complications and reoperations were collected using information documented in the medical records, including inpatient medical records, postoperative outpatient records, operative notes, and other medical notes. Surgical site complications were defined based on definitions of surgical site complications reported in the literature, including implant loosening, superficial and deep infections, knee stiffness, periprosthetic fractures, ligament injuries, hardware failure, and wound healing problems^13^. Reoperations were defined as any unscheduled operations resulting from surgical site complications, including implant revision, irrigation and debridement, open reduction internal fixation, wound revision, and others^14^.

### Statistical Analysis

Continuous variables with a normal distribution were described using mean ± standard deviation (SD), while median and interquartile range (IQR) were used for continuous variables with a skewed distribution. Categorical variables were described using frequency. Continuous variables were analyzed using independent sample *t*‐tests for those following a normal distribution, and non‐parametric tests were used for those without a normal distribution. Categorical variables were analyzed using the Pearson chi‐square or Fisher exact tests. A multivariable binary logistic regression model, with forward stepwise elimination (likelihood ratio), was used to explore the risk factors for surgical site complications and reoperations. The significance level was set at *P* < 0.05. All statistical analyses were conducted using SPSS 22.0 for Windows (IBM, Armonk, NY, USA).

## Results

### Intra‐operative Results

**Figure 1:**
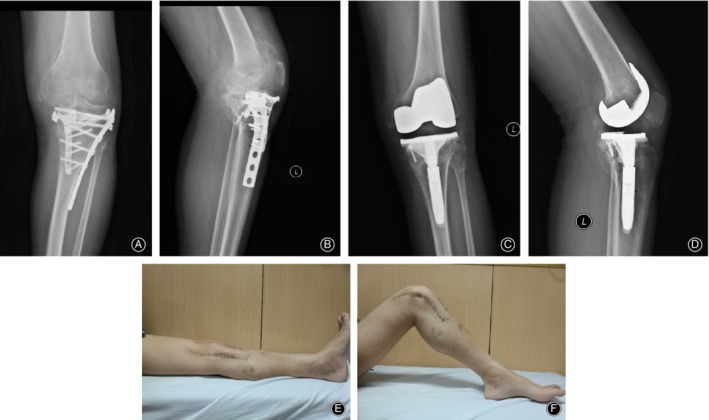
A 36‐year‐old male patient sustained a left tibial plateau fracture in a car accident and underwent open reduction and internal fixation at the age of 34. Radiographs showed advanced degenerative changes of the left knee, retained hardware, and a bone defect (A, B). Hardware removal and total knee arthroplasty (TKA) was performed with a long‐stemmed tibial component (C, D). Pain relief and satisfactory joint function was obtained. The ROM was 0°–90° at five‐year follow‐up (E, F).

**Figure 2:**
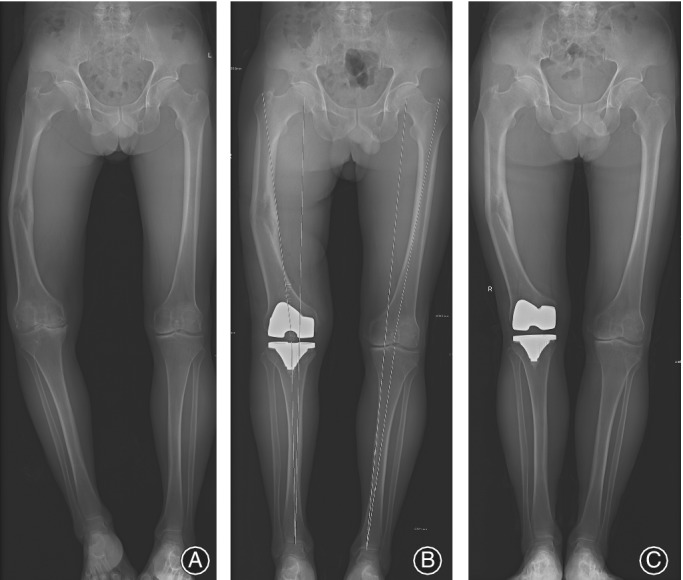
A 52‐year‐old female patient suffered from post‐traumatic osteoarthritis (PTOA) with a stiff knee after four operations due to nonunion of a femoral shaft fracture (A), and underwent total knee arthroplasty (TKA) with tibial tubercle osteotomies (TTOs) (B, C).

The median operative time was 110 (range, 80–140) minutes. One knee received a rectus snip and three knees received tibial tubercle osteotomies (TTOs) to facilitate surgical exposure. Hardware was completely removed in 14 knees (Fig. 1), while partial hardware removal was carried out in four knees. Navigation‐assisted TKA was performed in 10 knees (Fig. 2). Simultaneous femoral shaft osteotomy and TKA was performed in two knees for severe extra‐articular deformity.

### Clinical Outcomes

The median follow‐up period was 4.7 (range, 3.2–7.1) years. At the latest follow‐up, the median Knee Society knee score and function score improved from 35 (30,40) and 40 (30,50) to 90 (82,93) and 90 (65,100), respectively. The differences between them were significant (*P* < 0.001). The knee range of motion (ROM) increased from a median preoperative value of 90° (65°, 100°) to 110° (90°, 120°) at the latest follow‐up (*P* < 0.001). The degree of overall satisfaction after TKA surgery was very satisfied in 41 patients, satisfied in 20 patients, neutral in four patients, dissatisfied in four patients, and very dissatisfied in two patients. The overall satisfaction (very satisfied and satisfied) rate was 85.9% (61 knees), and the overall dissatisfaction (neutral, dissatisfied and very dissatisfied) rate was 14.1% (10 knees).

### Surgical Site Complications and Reoperations

Twelve of the 71 knees (16.9%) had 19 surgical site complications, including two infections, seven cases of stiffness, one periprosthetic fracture, five ligamentous injuries (three patellar tendon tears and two medial collateral ligament injuries), one hardware failure, and three wound healing problems (Table 2). Two cases of medial collateral ligament avulsion and one case of partial patellar tendon tear occurred intraoperatively, and were repaired directly during TKA surgery.

**Table 2:** Distribution of Number of Surgical Site Complications Based on Location of Prior Fracture

Complications	Location of prior fracture			Total
	Intra‐articular	Metaphysis	Diaphysis	
Superficial infection	0	0	1	1
Deep infection	0	0	1	1
Stiffness	1	3	3	7
Periprosthetic fracture	0	1	0	1
Patella tendon tear	0	2	1	3
Intra‐operative MCL injury	1	1	0	2
Hardware failure	0	0	1	1
Delayed wound healing	1	1	1	3
Total	3	8	8	19

MCL, medial collateral ligament.

Six knees (8.3%) underwent reoperation. One female patient underwent a debridement due to a superficial infection at the site of the TTO, 2 weeks after index TKA. Six months later, she suffered a patellar tendon tear and underwent cast immobilization for 2 months. At the 3‐year follow‐up, the implant was retained but the patient was dissatisfied with her knee function with 10° of ROM. Two patients underwent debridement due to delayed wound healing. Then the wounds healed without signs of infection. One patient fell down and suffered a supracondylar periprosthetic fracture 4 days after TKA. Open reduction and internal fixation was then performed, and the fracture healed eventually. One case of hardware failure occurred 3 months after the combined femoral shaft osteotomy and TKA, and was treated with extended internal fixation and autogenous bone grafting. A two‐stage revision was performed in one patient for the treatment of deep infection, which occurred 4 months after the primary TKA.

### Risk Factors for Surgical Site Complications and Reoperations

Univariate analysis revealed that patients in the surgical site complication group were more likely to have preoperative patella baja and longer operative times than those without complications (Table 3). Multivariate analysis revealed that preoperative patella baja was an independent risk factor for surgical site complication (OR = 6.860, 95% *CI*: 1.807–26.042, *P* = 0.005). Both univariate and multivariate analyses revealed that preoperative patella baja was an independent risk factor for reoperation (OR = 8.000, 95% *CI*: 1.319–48.538, *P* = 0.024).

**Table 3:** Univariate Analysis of Risk Factors for Surgical Site Complications

Variable	Surgical site complications		*P* value
	No (n = 59)	Yes (n = 12)	
Location of prior Fracture, n (%)			0.851
Metaphysis	16 (84.2%)	3 (15.8%)	
Diaphysis	28 (84.4%)	5 (15.2%)	
Intra‐articular	15 (78.9%)	4 (21.1%)	
Varus/valgus deformity, n (%)			0.584
≤10°	31 (86.1%)	5 (13.9%)	
11°~20°	20 (83.3%)	4 (16.7%)	
>20°	8 (72.7%)	3 (27.3%)	
Patella baja, n (%)			0.005*
No	49 (90.7%)	5 (9.3%)	
Yes	10 (58.8%)	7 (41.2%)	
Hardware retained, n (%)			0.485
No	45 (84.9%)	8 (15.1%)	
Yes	14 (77.8%)	4 (22.2%)	
Surgical history, n (%)			0.322
No	40 (87%)	6 (13%)	
Yes	19 (76%)	6 (24%)	
Stemmed components, n (%)			1.000
No	47 (82.5%)	10 (17.5%)	
Yes	12 (85.7%)	2 (14.3%)	
Preoperative ROM (°), median (IQR)	90.0 (70.0, 100.0)	77.5 (30.0, 100.0)	0.172
Age (years), mean ± SD	59.3 ± 10.7	58.4 ± 11.2	0.668
Operation time (minutes), median (IQR)	100.0 (75.0, 140.0)	132.5 (97.5, 177.5)	0.040*
Follow‐up period (years), median (IQR)	4.7 (3.3, 7.1)	4.4 (3.0, 7.6)	0.939

* denotes a significant difference between the two groups. ROM, range of motion.

## Discussion

Previous research has demonstrated favorable outcomes for TKA following fractures around the knee. Shearer *et al*.^8^ reported on 46 patients who underwent TKA for osteoarthritis secondary to femoral or tibial fracture with a follow‐up of 52 months; the knee score improved from 30 to 57 and the function score improved from 39 to 46. Abdel *et al*.^15^ reported on 45 patients who underwent TKA for osteoarthritis secondary to fracture of the tibial plateau with a follow‐up of 16 years, and the ROM improved from 99° to 105°, and the Knee Society knee score and function score improved from 44 and 52 to 80 and 70, respectively. The results of our study were similar to those observed in these studies. In our 71 cases, the mean ROM improved from 90° to 110° after TKA, and Knee Society knee score and function score improved from 35 and 40 to 90 and 90, respectively. The overall satisfaction (very satisfied and satisfied) rate was 85.9% (61 knees), which was comparable to our previously reported satisfaction rate (87.4%) after primary TKA in 748 patients in the same institution^12^. Thus, TKA is effective for the treatment of end‐stage PTOA and may result in satisfactory outcomes.

However, TKA in patients with PTOA is still challenging, and several special considerations should be kept in mind. Previous incisions performed for prior surgery increased the incidence of wound healing difficulties. When possible, the prior incision should be used to avoid skin necrosis between the incisions. If multiple incisions exist, one should choose the most lateral incision that can be used to protect the blood supply to the skin medially. Prior transverse incisions may be crossed with a longitudinal incision at a right angle. In rarely complex situations, wound closure may be difficult due to contracted scars, so a soft tissue expansion in advance or a simultaneous flap surgery should be considered^16^, ^17^. In some patients, angular deformity, canal sclerosis, or retained hardware associated with prior femoral fracture may hinder the use of an intramedullary rod for a distal femoral cut, while an extramedullary guide is less accurate and reproducible for femoral resection. In that case, a computer‐assisted navigation system could be used for TKA. Navigation‐assisted TKA provides an accurate bone cut and precise component positioning. Previous literature has reported excellent clinical outcomes with good alignment restoration and implant positioning^18^, ^19^, ^20^, ^21^. Severe ligamentous laxity or bone defects caused by intra‐articular fracture, and malalignment after malunion of an extra‐articular fracture, have created great challenges for soft tissue balancing in patients with PTOA. Revision due to instability occurred more frequently in TKAs performed due to previous fractures than in conventional TKAs^10^. Condylar constrained prostheses or even rotating hinged prostheses should be considered if adequate stability cannot be achieved. Purely intra‐articular bone defects could be addressed with a less‐constrained PS prosthesis with augments and long stems, which reduces the stress on the bone‐implant interface. Finally, knee joint stiffness was the most difficult problem to treat operatively, especially in cases with preoperative patella baja. The scar tissue and contracted extensor mechanism makes the surgical exposure quite difficult. The standard exposure technique is often insufficient, which creates a high risk of intraoperative patellar tendon rupture during knee flexion. Other techniques such as a rectus snip, lateral retinacular release, or TTO may be options to facilitate exposure. In spite of this, the incidence of poor postoperative ROM remains higher in those cases than in primary TKAs^1^.

Owing to compromise of the soft‐tissue envelope, poor bone stock, ligamentous instability, and multiple prior operations with or without retained hardware, the incidence of complications and revisions was substantially higher in TKA performed for PTOA than for primary arthritis. In previous literature, the complication rate after TKA for PTOA was 17.2%–31.3%, with complications including delayed wound healing, superficial or deep surgical site infections, stiffness of the knee, periprosthetic fracture, and deep venous thrombosis, while reoperation rates ranged from 13.8%–20.8%^1^, ^5^, ^22^, ^23^. In the present study, the complication rate was 16.9% and the reoperation rate was 8.3%, which was comparable to the results of previous studies. Meanwhile, most of the complications occurred at an early stage (within 3 months after surgery), which is also in accordance with other studies^9^, ^15^. The high complication rates would inevitably affect the survivorship of the prosthesis and functional outcome of patients after TKA, so it is important to identify related risk factors for TKA following femoral or tibial fractures.

Previous studies have reported several risk factors for unsatisfactory outcomes after TKA for PTOA. Shearer *et al*.^8^ noted that patients with combined tibial and femoral deformities and patients with soft‐tissue injuries experienced poor outcomes. Ge *et al*.^9^ reported that patients with previous fractures around the knee were more likely to experience surgical site complications and an increase in 90‐day readmission rate after conversion to TKA in contrast to patients with previous soft‐tissue injuries about the knee. El‐Galaly *et al*.^10^ reported an increased risk of early‐ and medium‐term TKA revision due to previous fractures of the distal femur and/or proximal tibia. Houdek *et al*.^1^ found that patients with a previous distal femoral fracture were at increased risk for postoperative complications (HR 1.47) compared with patients who had a history of proximal tibial fracture.

In our case series, patella baja was identified as an independent risk factor for surgical site complications and reoperations. Various methods had been utilized to quantify patella baja, including the Insall–Salvati (IS) ratio, modified IS (MIS) ratio, Blackburne–Peel (BP) ratio, and Caton–Deschamps (CD) ratio^24^, ^25^, ^26^. The Blackburne–Peel ratio was used to assess patellar height on weight‐bearing lateral radiographs while the knee was flexed approximately 30°, as previously described. Seil *et al*.^27^ and Berg *et al*.^28^ found this technique to be the most reproducible and accurate. Patella baja was found in 12 out of 71 of our knees (16.9%) by the Blackburne–Peel ratio. Multiple surgeries, prolonged immobilization, and anatomical distortions caused by fracture can all lead to patella baja.

The potential explanation for patella baja as a risk factor may be as follows. First, patella baja is usually accompanied by poor soft tissue conditions, which may increase the risk of incisional complications after TKA^29^. Second, surgical exposure of the knee can be quite difficult in patients with patella baja^30^, thereby increasing the risk of wound and extensor mechanism complications. In certain cases, the surgeon must perform a rectus snip or TTO as a result of severe patella baja. In our experience, TTO may provide a better surgical view for patients with severe patella baja. Although TTO is a demanding technique to improve the surgical approach during TKA, a straightforward surgical technique can lead to better results and minimize complication rates^31^. Third, patella baja can cause impingement between the inferior patella and the tibial plateau, thus restricting knee flexion^32^. Also, scar contracture caused by soft tissue injury or previous surgeries may result in stiffness of the knee^33^. Finally, soft tissue problems and a prolonged operative time due to patella baja may increase the incidence of infection after TKA. Above all, patella baja was a predictor of surgical site complications, reoperations, and poor clinical outcomes in the current study.

There were several limitations of our study. First, it was a retrospective study, which may weaken the strength of our conclusions. Second, the sample size was relatively small given the relative rarity of the pathological process that was studied. Thus, further research with a larger sample size is necessary. However, our study did have several strengths. To the best of our knowledge, this is the first study revealing the relationship between patella baja and increased surgical site complications and reoperations after TKA performed for PTOA. Second, it contained a homogeneous group of patients from a single institution, all of whom were operated on by surgeons with similar operative indications and surgical techniques. Our findings may help surgeons identify high‐risk patients, plan ahead, and take precautions to avoid related complications and reoperations.

### Conclusions

In summary, while TKA for PTOA can improve knee function significantly, the incidence of surgical site complications and reoperations is relatively high. Most of the complications occurred at an early stage. Preoperative patella baja was identified as a predictor of postoperative surgical site complications and reoperations. Surgeons should take precautions in the setting of patella baja if there is a TKA planned for PTOA.

#### Authorship declaration

All authors listed meet the authorship criteria according to the latest guidelines of the International Committee of Medical Journal Editors, and all authors are in agreement with the manuscript.
